# Iridoids and Flavonoids of Four Siberian Gentians: Chemical Profile and Gastric Stimulatory Effect

**DOI:** 10.3390/molecules201019172

**Published:** 2015-10-21

**Authors:** Daniil N. Olennikov, Nina I. Kashchenko, Nadezhda K. Chirikova, Larisa M. Tankhaeva

**Affiliations:** 1 Institute of General and Experimental Biology, Siberian Division, Russian Academy of Science, Sakh’yanovoy Str., 6, Ulan-Ude 670047, Russia; E-Mails: ninkk@mail.ru (N.I.K.); tankhaeva.l.m@gmail.ru (L.M.T.); 2 Department of Biochemistry and Biotechnology, North-Eastern Federal University, 58 Belinsky Str., Yakutsk 677027, Russia; E-Mail: hofnung@mail.ru

**Keywords:** *Gentiana algida*, *Gentiana decumbens*, *Gentiana macrophylla*, *Gentiana triflora*, iridoids, flavonoids, HPLC, gastric secretion stimulation

## Abstract

Some *Gentiana* species have been used by the nomadic people of Siberia as bitter teas or appetizers to eliminate digestive disorders (dyspepsia, heartburn, nausea, *etc.*). We studied the most frequently used gentians: *Gentiana algida*, *G. decumbens*, *G. macrophylla* and *G. triflora*. The aim of the present study was to evaluate the phytochemical features and gastrostimulatnt activity of these four gentian herbs. Five iridoids, seven flavones and mangiferin were detected in gentian herbs after analysis by microcolumn-RP-HPLC-UV-ESI-MS. A componential phytochemical profile of the *G. decumbens* herb is presented for the first time, as well as information about distinct phytochemicals found in gentian herbs. HPLC quantification of the specific compounds of gentian herbs demonstrated the high content of iridoids (24.73–73.53 mg/g) and flavonoids (12.92–78.14 mg/g). The results of biological activity evaluation of four gentian decoctions demonstrated their good ability to stimulate acid-, enzyme- and mucin-forming functions of the stomach attributed to mostly by iridoids and flavonoids. In general, it can be claimed that the gentian decoctions can be used as effective and safe appetizers and are also a good source of biologically active agents.

## 1. Introduction

**Figure 1:**
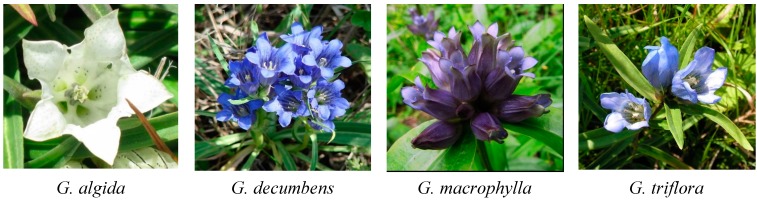
Gentians used in Siberia as bitter teas.

The basis of the nutritional system of Siberian people has traditionally consisted of products rich in animal protein and fat. Despite its profitable energy value, animal protein is a category of food with reduced digestibility, so excessive consumption leads to a variety of digestive disorders (dyspepsia, heartburn, nausea, *etc.*). To facilitate the process of digestion a variety of herbal products with a bitter taste was used [1,2,3]. The most commonly used herbal bitter species were gentianaceous plants that grow everywhere in the meadows and steppes of Siberia. These herbs are given uncomplicated names, like white or blue bitter herbs (plants with white or blue flowers), and their derivative beverages are referred to as *bitter teas* (*гашуун ундан*, Buryatian; *аhыы утах*, Yakutian; *йдарйичу умивўн*, Evenkian) [4]. Ethnopharmacological investigations allow us to identify the “bitter herbs” as various species of the genera *Halenia* [5,6,7,8], *Gentianopsis* [9], *Anagallidium* [10] and *Gentiana*. As part of an ongoing study of the chemistry and pharmacology of gentianaceous bitter teas, we present the results of analysis of the herbs of the four most frequently used gentians, including *G. algida* Pall. (cold gentian), *G. decumbens* L. f. (lying gentian), *G. macrophylla* Pall. (large-leaved gentian) and *G. triflora* Pall. (trianthous gentian) (Figure 1).

*G. algida* is a little gentian with white flowers which grows in crude marshy meadows of Central Siberia and the northwestern part of China. In Traditional Chinese Medicine (TCM) and Traditional Tibetan Medicine (TTM) practice this plant is used as a remedy for the treatment of pneumonia, throat diseases, infections and wounds [11,12]. Some phytochemicals have been discovered in *G. algida* herbs of Chinese origin, including iridoids, sterols, oleanolic acid, flavones, anofinic acid and fomannoxin [11,12,13,14,15]. The latter two compounds have demonstrated antifungal activity against *Cladosporium curcumerinum* [12]. *G. decumbens* is a typical plant of the steppefied meadows of all Siberian regions. The wide distribution of this species resulted in the most frequent application as a food plant in the form of tea, decoctions or infusions. TTM recommends *G. decumbens* for gastric diseases and some infections. To date there is no chemical data describing the phytochemical profile of *G. decumbens*. As for *G. macrophylla*, vegetating from Yakutian Siberia in the north to central China in the south, it is one of the most popular remedies in TCM, in which its roots are used to dispel rheumatism and ease pain [16]. Iridoids, triterpenes, sterols, and flavonoids have been isolated from roots of *G. macrophylla* [17]. The aerial part of this plant has been poorly investigated, although some iridoids and flavonoids from the flowers are known [18]. *G. triflora* is a large gentian with navy blue flowers which commonly grows in Siberian callows. In TTM the herb of *G. triflora* is used for the treatment of chronic stomach diseases, as it reduces gastric acidity, inflammation and cholecystitis [19]. The roots of *G. triflora* are used in TCM in many formulations for poor appetite and digestive problems. As in the case of *G. macrophylla*, the chemical compounds of *G. triflora* roots have been better studied than those of the herb. Only two flavones have been detected in *G. triflora* leaves—isoorientin and its 4′-*O*-glucoside [20]. To summarise known data devoted to these four gentian herbs (*G. algida*, *G. decumbens*, *G. macrophylla*, *G. triflora*), a poor general knowledge level of their chemical and pharmacological data can be confirmed. More characterisation is required of these plants that are widely used in everyday life.

In the present work, the herbs of *G. algida*, *G. decumbens*, *G. macrophylla*, *G. triflora* were submitted to analysis of specific classes of phytochemicals (iridoids, phenolic compounds). HPLC characterisation of a series of gentian herbs was conducted with the aim of quantifying phytochemicals. The influence of the decoctions of four gentian herbs on gastric secretion was also investigated.

## 2. Results and Discussion

### 2.1. Phytochemicals of Four Siberian Gentians Herbs

Phytochemicals of the *Gentianaceae* family include a wide spectrum of compounds with various chemical structures. Most common are iridoids, flavonoids, and xanthones [21]. Known data about phytochemicals of the *Gentiana* species included in the present investigation has indicated a low level of scrutiny of herb compounds in comparison to root constituents, e.g., about thirty compounds were isolated from *G. macrophylla* roots [16,17] *vs.* eight compounds discovered in *G. macrophylla* flowers [18]. Information on the chemical composition of *G. decumbens* is absent; therefore, in the first stage of the phytochemical study of the gentian teas we conducted an investigation of the *G. decumbens* herb.

#### 2.1.1. Phytochemicals of *G. decumbens* Herb

**Figure 2:**
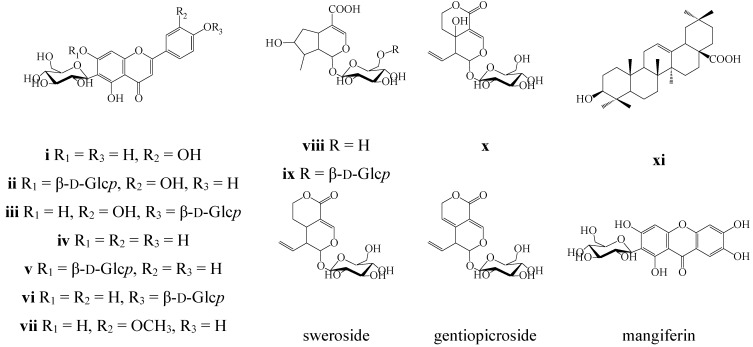
Phytochemicals, detected in four gentian herbs.

Seven flavonoids were isolated from 60%-MeOH extract of *G. decumbens* herb including isoorientin (luteolin-6-*C*-β-d-glucoside (**i**)), lutonarin (isoorientin-7-*O*-β-d-glucoside (**ii**)), isoorientin-4′-*O*-β-d-glucoside (**iii**)), isovitexin (apigenin-6-*C*-β-d-glucoside (**iv**)), saponarin (isovitexin-7-*O*-β-d-glucoside (**v**)), isosaponarin (isovitexin-4′-*O*-β-d-glucoside (**vi**)), and isoscoparin (3′-methoxyluteolin-6-*C*-β-d-glucoside (**vii**)). Also three iridoids, loganic acid (**viii**), loganic acid-6′-*O*-β-d-glucoside (**ix**), swertiamarin (**x**), as well as oleanolic acid (**xi**), were discovered in *G. decumbens* (Figure 2). Compounds were identified by comparison of their m.ps, optical rotation values, UV, ^1^H- and ^13^C-NMR spectroscopic and MS data with those reported in the literature and reference samples. This is the first report concerning to the chemical characterisation of *G. decumbens*.

It is known that flavonoids and iridoids are the characteristic metabolites from the *Gentianaceae* family. In particular, two biosynthetically related flavone *C*-glycosides, isoorientin (**i**) and isovitexin (**iv**), and two iridoids, loganic acid (**viii**) and swertiamarin (**x**), are by the far most common in the family [22]. Oleanolic acid (**xi**) is also a usual triterpene of gentianaceous plants [23].

The *O*-glycosides of **i** and **iv** have previously been isolated from some *Gentiana* species. Lutonarin (**ii**) has been determined in the *G. piasezkii* herb [24]. Isoorientin-4′-*O*-β-d-glucoside (**iii**) has been previously described in *G. affinis*, *G. algida*, *G. calycosa* [11], *G. macrophylla*, *G. straminea* [18] and *G. triflora* [20]. Saponarin (**v**) together with isoscoparin (**vii**) have been identified in *G. pneumonanthe* [25]. Isosaponarin (**vi**) has been detected in the roots of *G. linearis* [26]. The presence of loganic acid-6′-*O*-β-d-glucoside (**ix**) has only been described in the *Gentianaceae* species *G. rhodantha* [27].

#### 2.1.2. Identification of Iridoids and Phenolic Compounds of Herbs of Four Siberian Gentians

**Figure 3:**
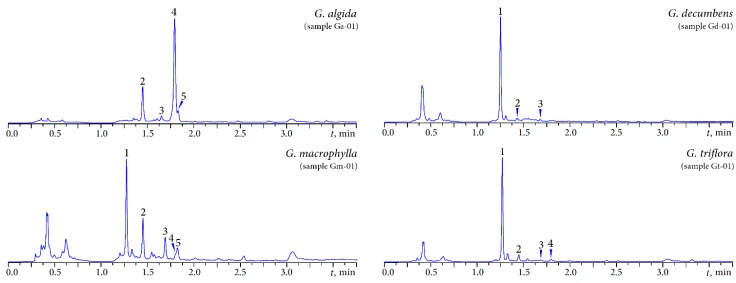
MC-RP-HPLC-UV chromatograms of the iridoid fractions of four gentian herbs detected at 230 nm. Compounds: **1**—loganic acid-6′-*O*-β-d-glucoside; **2**—loganic acid; **3**—swertiamarin; **4**—gentiopicroside; **5**—sweroside.

As a result of microcolumn (MC)-RP-HPLC-UV-ESI-MS analysis of the iridoid fractions of the gentian decoctions, five iridoid compounds were identified: loganic acid-6′-*O*-β-d-glucoside (*G. decumbens*, *G. macrophylla*, *G. triflora*), loganic acid, swertiamarin (all samples), gentiopicroside (*G. algida*, *G. macrophylla*, *G. triflora*) and sweroside (*G. algida*, *G. macrophylla*) (Figure 3, Table 1). The HPLC profile of the iridoid fraction of *G. algida* was different from the others since it showed a dominance of gentiopicroside and an absence of loganic acid-6′-*O*-β-d-glucoside. In contrast, the latter compound was prevalent in *G. decumbens*, *G. macrophylla*, and *G. triflora*, which had similar profiles.

Previous chemical investigations of the *G. algida* herb have led to isolation of the iridoids gentiopicroside [11], 1-*O*-β-d-glucopyranosylamplexine, trifloroside, rindoside, sweroside, 2′-(*o*,*m*-dihydroxybenzyl)sweroside [12], 6′-(2,3-dihydroxybenzyl)sweroside, and 6′-(2,3-dihydroxybenzyl) swertiamarin [13]. Loganic acid was detected in *G. algida* for the first time in this study. In flowers of *G. macrophylla* of Chinese origin, loganic acid, gentiopicroside, gentiopicroside-6′-*O*-β-d-glucoside, swertiamarin and sweroside have been discovered [18]. Loganic acid-6′-*O*-β-d-glucoside had not previously been described in *G. macrophylla*. Only the iridoid compound gentiopicroside was known as a component of the *G. triflora* herb [28], so loganic acid-6′-*O*-β-d-glucoside, loganic acid, and swertiamarin are newly identified compounds of *G. triflora*. The significance of loganic acid-6′-*O*-β-d-glucoside, found only in gentians of the *Pneumonanthe* (*G. triflora*) and *Aptera* sections (*G. decumbens*, *G. macrophylla*), may be discussed after additional investigation of other species of the mentioned sections.

**Table 1:** MC-RP-HPLC-UV-ESI-MS characterisation of the iridoids of four gentian herbs.

Peak No.	Compound	RT	Molecular Formula	Molecular Weight	λ_max_, nm	ESI-MS, *m*/*z*
1	Loganic acid-6′-*O*-β-d-glucoside ^a^	1.00	C_22_H_34_O_15_	538.19	235	561 [M + Na]^+^
2	Loganic acid ^b^	1.13	C_16_H_24_O_10_	376.36	234	399 [M + Na]^+^
3	Swertiamarin ^b^	1.28	C_16_H_22_O_10_	374.35	237	397 [M + Na]^+^
4	Gentiopicroside ^b^	1.42	C_16_H_20_O_9_	356.11	243, 272	379 [M + Na]^+^
5	Sweroside ^b^	1.43	C_16_H_22_O_9_	358.35	246	381 [M + Na]^+^

^a^ Compared with pure isolated compound (purity > 94%); ^b^ Compared with commercial standards.

**Figure 4:**
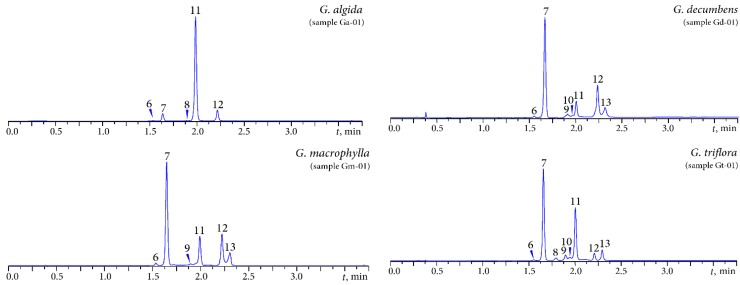
MC-RP-HPLC-UV chromatograms of the phenolic fractions of four gentian herbs detected at 334 nm. Compounds: **6**—lutonarin; **7**—isoorientin-4′-*O*-glucoside; **8**—mangiferin; **9**—saponarin; **10**—isosaponarin; **11**—isoorientin; **12**—isovitexin; **13**— isoscoparin.

The MC-RP-HPLC-DAD-ESI-MS procedure resulted in detection of eight phenolic compounds in the gentian herbs (Figure 4, Table 2). Lutonarin, isoorientin-4′-*O*-glucoside, saponarin, isoorientin, and isovitexin were discovered in all samples. The presence of isosaponarin was marked in *G. decumbens* and *G. triflora* and of isoscoparin in *G. decumbens*, *G. macrophylla* and *G. triflora*. Xanthone *C*-glycoside was found only in *G. triflora*.

According to earlier data, isoorientin, isoorientin-4′-*O*-glucoside [11], orientin [12], isoscoparin [14], swertisin, and swertijaponin [15] have been identified in the herb of *G. algida*. Lutonarin, saponarin and isovitexin were detected in *G. algida* for the first time. Isoorientin-4′-*O*-glucoside, isoorientin and vitexin have previously been described as components of *G. macrophylla* flowers [18], but lutonarin, saponarin, isovitexin and isoscoparin had not previously been found in this species.

Two flavones (isoorientin-4′-*O*-glucoside and isoorientin) had previously been described in the *G. triflora* herb [20]; however, lutonarin, mangiferin, saponarin, isovitexin and isoscoparin are newly identified compounds of this plant. Of particular note is the discovery of 1,3,6,7-tetrahydroxyxanthone 2-*C*-glucoside (mangiferin) in *G. triflora*. This compound occurs sporadically in the *Gentianaceae* family and is often found with *C-*glycosylflavones [22] and has previously been described in the other members of the *Pneumonanthe* section of *Gentianaceae* family which includes *G. triflora*; these are *G. asclepiadea* [29] and *G. pneumonanthe* [11].

**Table 2:** MC-RP-HPLC-DAD-ESI-MS characterisation of the phenolic compounds of four gentian herbs.

Peak No.	Compound	RT	Molecular Formula	Molecular Weight	λ_max_, nm	ESI-MS, *m*/*z*
6	Lutonarin ^a^	1.00	C_27_H_29_O_16_	609.52	256, 270, 348	611 [M + H]^+^, 449 [(M + H) − 162]^+^
7	Isoorientin-4′-*O*-glucoside ^a^	1.06	C_27_H_29_O_16_	609.52	270, 337	611 [M + H]^+^, 449 [(M + H) − 162]^+^
8	Mangiferin ^b^	1.16	C_19_H_18_O_11_	422.35	240, 258, 317, 361	423 [M + H]^+^
9	Saponarin ^b^	1.24	C_27_H_30_O_15_	594.16	272, 334	595 [M + H]^+^, 433 [(M + H) − 162]^+^
10	Isosaponarin ^b^	1.26	C_27_H_30_O_15_	594.16	274, 325	595 [M + H]^+^, 433 [(M + H) − 162]^+^
11	Isoorientin ^b^	1.30	C_21_H_20_O_11_	448.39	260, 269, 349	449 [M + H]^+^
12	Isovitexin ^b^	1.42	C_21_H_20_O_10_	432.39	270, 337	433 [M + H]^+^
13	Isoscoparin ^a^	1.49	C_22_H_21_O_11_	461.11	256, 270, 352	462 [M + H]^+^

^a^ Compared with pure isolated compounds (purity > 94%); ^b^ Compared with commercial standards.

### 2.2. HPLC Quantification of the Iridoids and Phenolic Compounds of Four Gentian Herbs

Quantification of the main phytochemicals in the gentian herbs was realised by an MC-HPLC-UV procedure previously applied for analysis of the *Anagalidium dichotomum* herb [10]. Of the 12 compounds including five iridoids, six flavone-*C*/*O*-glycosides and mangiferin, from eight to 11 analytes were found in detectable levels in 16 samples of the gentian herbs. All the contents are summarised in Table 3.

**Table 3:** Content of iridoids, flavonoids and mangiferin in four gentian herbs, mg/g (±SD) dry plant weight.

Compound	*G. algida* (Ga)				*G. decumbens* (Gd)			
	Ga-01	Ga-02	Ga-03	Ga-04	Gd-01	Gd-02	Gd-03	Gd-04
**Iridoids**								
**1**	12.32 ± 0.25	10.63 ± 0.21	9.67 ± 0.18	15.93 ± 0.31	5.37 ± 0.10	4.31 ± 0.09	6.07 ± 0.12	3.81 ± 0.07
**2**	n.d.	n.d.	n.d.	n.d.	54.81 ± 1.04	61.37 ± 1.22	57.34 ± 1.14	51.37 ± 1.02
**3**	3.19 ± 0.05	1.95 ± 0.03	2.73 ± 0.05	2.49 ± 0.04	tr.	tr.	tr.	tr.
**4**	2.54 ± 0.04	4.09 ± 0.07	2.09 ± 0.03	7.80 ± 0.16	n.d.	n.d.	n.d.	n.d.
**5**	27.71 ± 0.58	33.84 ± 0.71	45.87 ± 0.96	29.37 ± 0.52	n.d.	n.d.	n.d.	n.d.
**Subtotal**	45.76	50.51	60.36	55.59	60.18	65.68	63.41	55.18
**Flavonoids**								
**7**	1.53 ± 0.03	0.82 ± 0.01	1.67 ± 0.03	1.18 ± 0.02	6.37 ± 0.10	7.50 ± 0.15	9.11 ± 0.19	6.81 ± 0.12
**9**	3.16 ± 0.05	1.82 ± 0.03	0.94 ± 0.01	3.84 ± 0.06	1.93 ± 0.03	0.94 ± 0.01	1.98 ± 0.04	2.07 ± 0.03
**10**	n.d.	n.d.	n.d.	n.d.	tr.	tr.	tr.	tr.
**11**	21.31 ± 0.34	29.57 ± 0.47	39.53 ± 0.75	21.18 ± 0.38	1.93 ± 0.03	1.94 ± 0.03	2.84 ± 0.05	4.14 ± 0.07
**12**	4.42 ± 0.08	6.11 ± 0.10	2.68 ± 0.04	6.73 ± 0.12	2.69 ± 0.04	1.64 ± 0.03	2.68 ± 0.05	3.24 ± 0.06
**13**	n.d.	n.d.	n.d.	n.d.	1.32 ± 0.02	0.92 ± 0.01	1.11 ± 0.02	1.53 ± 0.02
**Subtotal**	30.42	38.32	44.82	32.93	14.24	12.94	17.72	17.79
**Xanthones**								
**8**	n.d.	n.d.	n.d.	n.d.	n.d.	n.d.	n.d.	n.d.
**Subtotal**	n.d.	n.d.	n.d.	n.d.	n.d.	n.d.	n.d.	n.d.
**Total**	76.18	88.83	105.18	88.52	74.42	78.62	81.13	72.97
**Compound**	***G. macrophylla* (Gm)**				***G. triflora* (Gt)**			
	**Gm-01**	**Gm-02**	**Gm-03**	**Gm-04**	**Gt-01**	**Gt-02**	**Gt-03**	**Gt-04**
**Iridoids**								
**1**	6.37 ± 0.12	7.21 ± 0.12	6.37 ± 0.14	7.83 ± 0.14	4.12 ± 0.08	3.21 ± 0.06	2.32 ± 0.04	5.31 ± 0.09
**2**	15.93 ± 0.31	19.34 ± 0.41	19.02 ± 0.34	24.09 ± 0.45	63.18 ± 1.20	61.23 ± 1.10	66.81 ± 1.20	65.92 ± 1.31
**3**	0.94 ± 0.01	0.54 ± 0.01	0.64 ± 0.01	0.27 ± 0.00	tr.	tr.	tr.	tr.
**4**	0.37 ± 0.00	0.30 ± 0.00	0.51 ± 0.01	0.31 ± 0.00	n.d.	n.d.	n.d.	n.d.
**5**	1.12 ± 0.01	1.02 ± 0.01	0.92 ± 0.01	0.63 ± 0.01	2.83 ± 0.05	3.48 ± 0.07	2.57 ± 0.05	2.30 ± 0.04
**Subtotal**	24.73	28.41	27.46	33.13	70.13	67.92	71.70	73.53
**Flavonoids**								
**7**	15.38 ± 0.29	15.74 ± 0.25	19.32 ± 0.34	12.81 ± 0.23	22.67 ± 0.36	40.27 ± 0.84	35.94 ± 0.61	32.12 ± 0.57
**9**	1.02 ± 0.01	1.46 ± 0.02	0.63 ± 0.01	0.19 ± 0.00	4.06 ± 0.07	2.84 ± 0.04	3.19 ± 0.04	2.02 ± 0.03
**10**	n.d.	n.d.	n.d.	n.d.	3.87 ± 0.07	1.16 ± 0.02	0.57 ± 0.01	2.53 ± 0.03
**11**	4.30 ± 0.07	5.61 ± 0.11	7.11 ± 0.14	6.91 ± 0.12	14.25 ± 0.24	19.94 ± 0.35	31.16 ± 0.59	16.96 ± 0.30
**12**	6.84 ± 0.12	4.20 ± 0.07	7.08 ± 0.14	6.63 ± 0.11	1.73 ± 0.03	2.31 ± 0.04	5.22 ± 0.11	1.37 ± 0.02
**13**	1.69 ± 0.02	2.64 ± 0.04	2.47 ± 0.05	1.37 ± 0.02	3.51 ± 0.06	2.26 ± 0.03	2.06 ± 0.02	4.19 ± 0.08
**Subtotal**	29.23	29.65	36.61	27.91	50.09	68.78	78.14	59.19
**Xanthones**								
**8**	n.d.	n.d.	n.d.	n.d.	2.14 ± 0.04	3.71 ± 0.08	1.97 ± 0.03	4.18 ± 0.08
**Subtotal**	n.d.	n.d.	n.d.	n.d.	2.14	3.71	1.97	4.18
**Total**	53.96	58.06	64.07	61.04	122.36	140.41	151.81	136.90

**1**—loganic acid; **2**—loganic acid-6′-*O*-β-d-glucoside; **3**—swertiamarin; **4**—sweroside; **5**—gentiopicroside; **7**—isoorientin-4′-*O*-glucoside; **8**—mangiferin; **9**—saponarin; **10**—isosaponarin; **11**—isoorientin; **12**—isovitexin; **13**—isoscoparin; n.d.—not detected (<limit of detection); tr.—traces amounts (<limit of quantification).

The results showed that the total content of quantifiable compounds in the gentian herb samples was 53.96–64.07 mg/g in *G. macrophylla* herb, 72.97–81.13 mg/g in *G. decumbens* herb, 76.18–105.18 mg/g in *G. algida* herb, and 122.36–151.81 mg/g in *G. triflora* herb. The highest iridoids content, 67.92–73.53 mg/g, was detected in *G. triflora* herb which dominate compound was loganic acid-6′-*O*-β-d-glucoside (61.23–66.81 mg/g). The prevalence of this component observed in *G. decumbens* herb (51.37–61.37 mg/g) and *G. macrophylla* herb (15.93–24.09 mg/g). Gentiopicroside was a main iridoid of *G. algida* herb containing it as 27.71–45.87 mg/g. Detectable amounts of sweroside and swertiamarin were found in *G. algida* (2.09–7.80 mg/g and 1.95–3.19 mg/g) and *G. macrophylla* herbs (0.30–0.51 mg/g and 0.27–0.94 mg/g). The maximal concentration of flavonoids was in *G. triflora* (50.09–78.14 mg/g), an herb with a highest content of isoorientin-4′-*O*-glucoside (22.67–40.27 mg/g). The *G. algida*, unlike of other species, contained the high level of isoorientin (21.18–39.53 mg/g) which content in other herbs was 1.93–4.14 mg/g in *G. decumbens*, 4.30–7.11 mg/g in *G. macrophylla*, and 14.25–31.16 mg/g in *G. triflora*. The contents of isovitexin and saponarin were on the quantifiable levels in all species reaching 1.37–7.08 mg/g and 0.19–4.06 mg/g, respectively. The measurable amounts of isosaponarin were discovered only in *G. triflora* herb (0.57–3.87 mg/g). Contents of isoscoparin in gentian herbs were 0.92–1.53 mg/g in *G. decumbens*, 1.37–2.64 mg/g in *G. macrophylla*, 2.06–4.19 mg/g in *G. triflora*, and undetectable in *G. algida*. The presence of the discovered xanthone mangiferin was observed in *G. triflora* herb only (1.97–3.71 mg/g).

To summarize the results of HPLC quantification of the specific compounds of four gentians it should be concluded that the herbs of *G. algida*, *G. decumbens*, *G. macrophylla* and *G. triflora* are the good source of iridoids and flavonoids.

### 2.3. Influence of the Gentian Decoctions and Individual Compounds on Gastric Secretory Function

**Figure 5:**
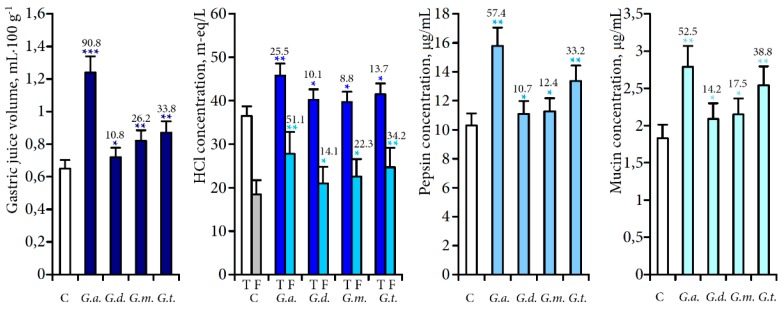

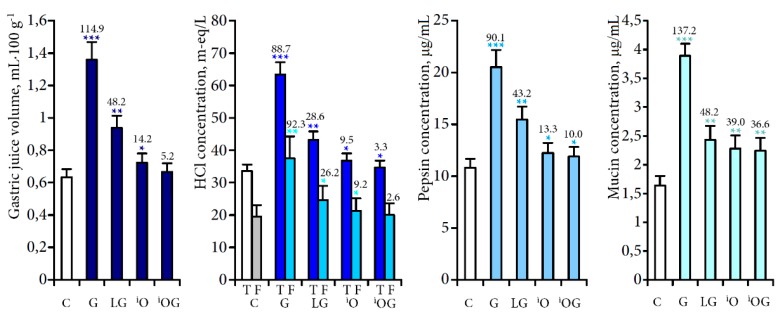
Influence of decoctions of four gentian herbs and individual compounds on gastric juice parameters in rats. Under bars: percentage difference compared to the control. Values shown are mean ± SD (*n* = 10–12). Asterisks indicate statistically significant values from control (* *p* < 0.05; ** *p* < 0.01; *** *p* < 0.001). C—control, *G.a.*—*G. algida*, *G.d.*—*G. decumbens*, *G.m.*—*G. macrophylla*, *G.t.*—*G. triflora*, G—gentiopicroside, LG—loganic acid-6′-*O*-glucoside, ^i^O—isoorientin, ^i^OG—isoorientin-4′-*O*-glucoside; T—total HCl content, F—free HCl content.

Oral intragastral administration of the gentian herb decoctions in a dose of 100 mg/kg stimulated acid- and enzyme-forming functions of the stomach (Figure 5). In all experimental groups an increase of gastric juice volume, total and free HCl concentration as well as pepsin concentration was observed. Maximal output of gastric juice volume (90.8% compared to the control group), total and free HCl concentration (25.5% and 51.1%) and pepsin concentration (57.4%) was observed in the *G. algida* group. *G. triflora* decoction was characterised by a medium increase of all the parameters of stomach secretion, while *G. decumbens* and *G. macrophylla* preparations demonstrated respectively weak activity.

Among individual compounds which were applied in a dose of 20 mg/kg, gentiopicroside had the most pronounced stimulation activity on gastric juice secretion (114.9% compared to the control group), both total and free HCl concentrations (88.7% and 92.3%) and pepsin production (90.1%). Loganic acid-6′-*O*-glucoside demonstrated a less pronounced influence on the secretory parameters of the stomach; however, the differences in the results obtained compared to the control group allow us to describe it as a stimulator with medium effectiveness. Flavone-*C*-glucoside (isoorientin) and its *O*-derivative (isoorientin-4′-*O*-glucoside) had the weakest activity, confirming the leading role of iridoids for stomach secretion stimulation.

Discussing the possible reasons for the observed differences it should be mentioned that the main chemical feature of *G. algida* decoction is a high gentiopicroside content. This iridoid compound is characterised as the most bitter that suppose it has the most pronounced stimulation action on digestive secretions by activating a trigger system of the digestive tract. Meanwhile, despite *G. decumbens*, *G. macrophylla*, and *G. triflora* decoctions were less bitter, we can describe them as gastric stimulants although with a less pronounced effect. Interestingly, although the main role of gentiopicroside and gentiopicroside-containing remedies for stimulation of gastric secretion was discussed previously [30], this ability of the loganic acid derivative is demonstrated in this paper for the first time.

The mucin concentration in stomach juice is another important indicator of stomach secretory activity. Mucin is an important protective factor for the gastric mucosa and consists of water and glycoproteins that cover the entire gastrointestinal mucosa. It is capable of acting as an antioxidant, and thus can reduce mucosal damage mediated by oxygen free radicals [31]. In this study, an increase of mucin secretion (14.2%–52.5% compared with the control) was observed in rats administered with the gentian teas, indicating the ability of the mucosal membrane to protect the mucosa from physical damage, irritant stomach secretions (HCl and pepsin) and back-diffusion of hydrogen ions.

All the phytochemicals studied influence mucin secretion positively with the highest effectiveness for gentiopicroside (137.2% compared with the control). The values of stimulation of loganic acid-6′-*O*-glucoside, isoorientin and isoorientin-4′-*O*-glucoside were close (36.6%–48.2%) and significantly higher than in the control but 2.8–3.8 times lower than the same parameter of gentiopicroside.

Generally, this study proved stimulant activity of the decoctions of four gentian herbs on the secretory functions of the stomach which is due to iridoids as well as flavonoids. The fact of simultaneous enriching of stomach juice by HCl, pepsin and mucin allows characterising of gentian decoctions as good and safe stimulators of gastric function.

Summarising the research, the striking insight of the nomadic people of Siberia in empirically identifying the positive effects of certain plant products on the human body evokes admiration. As a result of numerous investigations they managed to find the relationship between consumption of bitter herbal teas (mostly gentianaceous plants) with a consequent improvement of the digestion process. Certainly they could not explain the true nature of the impact of bitter tea on humans, but empirical knowledge gained during evolution was the basis for the introduction to modern customs the positive tradition of drinking bitter appetizers before eating. As a result of our study we are able to identify the cause of the use of bitter gentian teas as appetizers. The decoctions of the four species studied (*G. algida*, *G. decumbens*, *G. macrophylla*, *G. triflora*) are characterised by their high content of bioactive phytochemicals. In particular, the iridoids show a predominant influence on the secretion of digestive juices in the stomach and thus promote the process of digestion. Generally, *G. algida*, *G. decumbens*, *G. macrophylla* and *G. triflora* herbs could be promising candidates for further studies designed to obtain more evidence on their components with potential gastroprotective and digestive activity.

## 3. Experimental Section

### 3.1. General

#### 3.1.1. Chemicals, Sorbents

Extrasynthese (Lyon, France)—isoorientin (Cat. No. 1055 S, ≥99%), isovitexin (Cat. No 1235 S, ≥99%), loganic acid (Cat. No 0230 S, ≥99%), saponarin (Cat. No 1238 S, ≥98%); GE Healthcare (Little Chalfont, UK)—Sephadex^®^ LH-20 (Cat. No GE17-0090-01); Sigma-Aldrich (St. Louis, MO, USA)—Amberlite^®^ XAD7HP (Cat. No XAD7, 20–60 mesh), anthrone (Cat. No 319899, ≥97%), lithium perchlorate (Cat. No 431567, ≥99.99%), mangiferin (Cat. No 06279, ≥98%), octadecylfunctionalized silica gel (RP-Si0_2_; Cat. No 377635, 200–400 mesh), perchloric acid (Cat. No 311421, ≥70%, 99.999% trace metals basis), polyamide for CC (Cat. No 02395), silica gel (Si0_2_; Cat. No 236810, Davisil Grade 643, pore size 150 Å, 200–425 mesh), sweroside (Cat. No SMB00083, ≥95%), swertiamarin (Cat. No SMB0008O, ≥95%); Wuhan ChemFaces Biochemical Co., Ltd. (Wuhan, China)—isosaponarin (Cat. No CFN9O 133, ≥95%).

#### 3.1.2. Equipment

UV-Vis spectrophotometry—SF-2000 UV-Vis-spectrophotometer (OKB Specter, St. Peterburg, Russia); Melting point—IA9100 micromelting point apparatus (Thermo Scientific, Waltham, MA, USA); NMR spectroscopy—VXR 500S spectrometer (Varian, Palo Alto, CA, USA); ESI-MS—LCQ Classic LC/MS/MS mass spectrometer (Thermo Scientific); prep. HPLC-UV—Summit system with UV-Vis detector (Dionex, Sunnyvale, CA, USA), LiChrosorb RP-18 (4.6 × 250 mm, 5 μm, Supelco, Bellefonte, PA, USA), T = 35 °C, flow rate 1 mL/min; analyt. MC-HPLC—microcolumn chromatograph Econova MiLiChrom A-02 (Novosibirsk, Russia).

### 3.2. Plant Material

The samples of four gentian species were collected in the flowering period in the Siberian Regions (Russian Federation—Buryatia Republic (BR), Sakha (Yakutia) Republic (SR), Irkutsk Region (IR)): *G. algida*—Bagdarin (Bauntovskii region, BR, 12.VII.2014, 54°28′26″ N, 113°29′16″ E, voucher specimen No. Gn/h-62/04-11/0714; sample Ga-01); Orlik (Okinskii region, BR, 24.VII.2014, 52°34′30″ N, 99°43′28″ E, voucher specimen No. Gn/h-106/04-18/0714; sample Ga-02); Vydrino (Kabanskii region, BR, 25.VII.2012, 51°12′19″ N, 104°24′43″ E, voucher specimen No. Gn/h-25/04-72/0712; sample Ga-03); Irkhidey (IR, 14.VII.2013, 53°27′57″ N, 103°39′55″ E, voucher specimen No. Gn/h-03/04-12/0713; sample Ga-04); *G. decumbens*—Mukhorshibir’ (Mukhorshibirskii region, BR, 10.VII.2014, 51°2′14″ N, 107°55′31″ E, voucher specimen No. Gn/h-31/09-24/0714; sample Gd-01); Tarbagatai (Tarbagataiskii region, BR, 27.VII.2013, 51°27′24″ N, 107°24′4″ E, voucher specimen No. Gn/h-16/02-15/0713; sample Gd-02); Uro (Barguzinskii region, BR, 16.VII.2012, 53°32′43″ N, 109°53′58″ E, voucher specimen No. Gn/h-31/11-61/0712; sample Gd-03); Solyanka (Olyokminskii ulus, SR, 20.VII.2012, 60°26′52″ N, 120°45′53″ E, voucher specimen No. Gn/h-16-Y/04-31/0712; sample Gd-04); *G. macrophylla*—Turka (Pribaikal’skii region, BR, 16.VII.2014, 52°55′46″ N, 108°16′31″ E, voucher specimen No. Gn/h-71/06-12/0714; sample Gm-01); Orongoy (Ivolginskii region, BR, 22.VII.2014, 51°32′1″ N, 107°0′39″ E, voucher specimen No. Gn/h-17/15-11/0714; sample Gm-02); Alla (Kurumkanskii region, BR, 10.VII.2013, 54°40′0″ N, 110°50′20″ E, voucher specimen No. Gn/h-32/07-16/0713; sample Gm-03); Yakutsk (SR, 18.VII.2012, 62°2′5″ N, 129°31′42″ E, voucher specimen No. Gn/h-09-Y/02-11/0712; sample Gm-04); *G. triflora*—Posol’skoye (Kabanskii region, BR, 25.VII.2014, 52°0′20″ N, 106°11′43″ E, voucher specimen No. Gn/h-63/04-09/0714; sample Gt-01); Romanovka (Yeravninskii region, BR, 26.VII.2014, 53°11′53″ N, 112°45′16″ E, voucher specimen No. Gn/h-21/04-10/0714; sample Gt-02); Nyuya (Lenskii ulus, SR, 12.VII.2013, 60°34′57″ N, 116°18′10″ E, voucher specimen No. Gn/h-03-Y/02-07/0713; sample Gt-03); Mirniy (Mirninskii ulus, SR, 15.VII.2012, 62°30′11″ N, 113°54′50″ E, voucher specimen No. Gn/h-07-Y/11-22/0712; sample Gt-04).

### 3.3. Extraction and Isolation of Phytochemicals from G. decumbens Herb

Air-dried, ground herb of *G. decumbens* (sample Gd-01; 5.5 kg) were extracted with 60% EtOH at 50 °C, three times for 4 h each, and the combined extracts were partitioned with CHCl_3_ and *n*-BuOH to yield 143 and 1408 g of CHCl_3_ (Gd-F_1_) and *n*-BuOH fractions (Gd-F_2_) respectively. The Gd-F_1_ fr. (50 g) was chromatographed using flash chromatography on a silica column (3 × 100 cm, CHCl_3_-MeOH (100:0→0:100)), to yield 44.1 g of oleanolic acid (**xi**) [32]. The Gd-F_2_ fr. (900 g) was loaded on Amberlite XAD7HP (4 kg) and eluted water-MeOH mixt. (100:0→30:70→60:40→0:100) to give 4 sub-fractions Gd-F_2_-1 (235 g), Gd-F_2_-2 (204 g), Gd-F_2_-3 (95 g) and Gd-F_2_-4 (24 g). Subfr. Gd-F_2_-1 (200 g) was chromatographed by 20 g portions on a Sephadex LH-20 column (3 × 100 cm, MeOH-H_2_O (100:0→50:50)) following prep. HPLC (mobile phase A—water, mobile phase B—acetonitrile; gradient programme: 0–120 min, 0%–10% B; detector wavelength—254 nm) to give finally 16.5 g of loganic acid (**viii**), 37.2 g of loganic acid-6′-*O*-β-d-glucoside (**ix**) [33] and 421 mg of swertiamarin (**x**) [34]. Subfr. Gd-F_2_-2 (100 g) was chromatographed on polyamide column [5 × 80 cm, H2O–MeOH (100:0→40:60)] and a Sephadex LH-20 column (2 × 100 cm, MeOH–H_2_O (100:0→30:70)) to yield 57 mg of lutonarin (isoorientin-7-*O*-β-d-glucoside (**ii**)) [35], 14.6 g of isoorientin-4′-*O*-β-d-glucoside (**iii**) [36], 1.4 g of saponarin (isovitexin-7-*O*-β-d-glucoside (**v**)) [37], and 27 mg of isosaponarin (isovitexin-4′-*O*-β-d-glucoside (**vi**)) [36]. Subfr. Gd-F_2_-3 (80 g) was repeatedly chromatographed on a Sephadex LH-20 column (2 × 100 cm, MeOH–H_2_O (100:0→50:50)) and RP-SiO_2_ column (2 × 40 cm, H_2_O–MeCN (100:0→40:60)) to yield 4.3 g of isoorientin (luteolin-6-*C*-β-d-glucoside; **i**), 3.8 g of isovitexin (apigenin-6-*C*-β-d-glucoside (**iv**)) [38], and 37 mg of isoscoparin (3′-methoxyluteolin-6-*C*-β-d-glucoside (**vii**)) [25].

*Loganic acid-6′-O-*β*-d-glucoside* (**ix**): Off-white powder. UV (λ_max_) nm 235. (+)ESI-MS *m*/*z* 561 [M + Na]^+^. ^1^H-NMR (500 MHz, MeOH-*d*_4_) δ 1.07 (3H, d, *J* = 7.0 Hz; H-10), 1.68 (1H, ddd, *J* = 14.2, 8.1, 5.1 Hz; H-6_B_), 1.92 (1H, m; H-8), 2.15 (1H, ddd, *J* = 14.2, 8.1, 1.2 Hz; H-6_A_), 2.05 (1H, m; H-9), 3.02 (1H, qd, *J* = 8.1, 1.0 Hz; H-5), 3.16 (1H, dd, *J* = 8.0 Hz; H-2′, 1-O-Glcp), 3.20 (1H, dd, *J* = 9.0, 8.0 Hz; H-2′′, 6′-O-Glcp), 3.25–3.44 (4H, m; H-3′, H-4′, 1-O-Glcp; H-3′′, H-4′′, 6′-O-Glcp), 3.49 (1H, m; H-5′′, 6′-O-Glcp), 3.52 (1H, m; H-5′, 1-O-Glcp), 3.63 (1H, dd, *J* = 11.5, 6.0 Hz; H-6′′_B_, 6′-O-Glcp), 3.81 (1H, dd, *J* = 11.5, 2.0 Hz; H-6′′_A_, 6′-O-Glcp), 3.94 (1H, dd, *J* = 12.0, 5.0 Hz; H-6′_B_, 1-O-Glcp), 4.10 (1H, dd, *J* = 12.0, 1.5 Hz; H-6′_A_, 1-O-Glcp), 4.19 (1H, td, *J* = 5.1, 2.1 Hz; H-7), 4.64 (1H, d, *J* = 8.0 Hz; H-1′, 1-O-Glcp), 4.81 (1H, d, *J* = 8.0 Hz; H-1′′, 6′-O-Glcp), 5.24 (1H, d, *J* = 5.0 Hz; H-1), 7.21 (1H, d, *J* = 1.0 Hz; H-3). ^13^C-NMR (125 MHz, MeOH-d_4_) δ 14.5 (CH_3_, C-10), 32.7 (CH, C-5), 42.5 (CH, C-8), 43.4 (CH_2_, C-6), 47.2 (CH, C-9), 62.7 (CH_2_, C-6′′, 6′-O-Glcp), 70.2 (CH_2_, C-6′, 1-O-Glcp), 71.5 (CH, C-4′′, 6′-O-Glcp), 71.9 (CH, C-4′, 1-O-Glcp), 74.1 (CH, C-2′, 1-O-Glcp), 74.6 (CH, C-2′′, 6′-O-Glcp), 76.4 (CH, C-3′, 1-O-Glcp), 77.0 (CH, C-3′′, 6′-O-Glcp), 77.5 (CH, C-7), 78.2 (CH, C-5′, 1-O-Glcp), 78.4 (CH, C-5′′, 6′-O-Glcp), 98.4 (CH, C-1), 101.0 (CH, C-1′, 1-O-Glcp), 104.9 (CH, C-1′′, 6′-O-Glcp), 114.2 (CH, C-4), 152.6 (CH, C-3).

### 3.4. Polyamide Solid Phase Extraction (SPE) of Iridoids and Flavonoids

The sample of dried milled herb (1 g) was added to distilled water (100 mL), heated on heater plate and boiled 10 min. The mixture was left to stand at room temperature for 15 min, and than filtered under reduced pressure. A polyamide column (1.5 g) was prepared, primed with methanol (40 mL) followed by tridistilled water (td-water, 80 mL). An aliquot (1 mL) of gentian decoction was loaded on the column. Sequential elution was done with 30 mL of td-water (iridoids fraction) and 70% ethanol (phenolic fraction). The fractions after SPE extraction were concentrated to 1 mL, filtered through a 0.22-μm PTFE syringe filter before HPLC separation and stored at −10 °C prior analysis.

### 3.5. MC-RP-HPLC-UV-ESI-MS and MC-HPLC-UV Quantification Conditions

MC-RP-HPLC-UV-ESI-MS experiments were performed on an Econova MiLiChrom A-02 microcolumn chromatograph coupled with UV- and ESI-MS-detectors, using ProntoSIL-120-5-C18 AQ column (1 × 50 mm, Ø 1 μm; Metrohm AG; Herisau, Switzerland), column temperature was 35 °C. Eluent A was 0.2 М LiClO_4_ in 0.006 M HClO_4_ and eluent B was acetonitrile. The injection volume was 1 μL, and elution was at 600 μL/min. Gradient program: 0–2.5 min, 5%–35% B; 2.5–4 min, 35%–70% B. UV-detector wavelengths were 254 nm (iridoids) and 334 nm (flavonoids, mangiferin). UV-spectra were recorded at the range 190–400 nm. The eluate was introduced into mass spectrometer without splitting. The TOF mass spectrometer Agilent 6200 TOF LC/MS (Santa Clara, CA, USA) with ESI interface was used. The parameters of ESI source were: nebulizer pressure 40 psi; nebulizing gas and drying gas were nitrogen at a flow 10 L∙min^−1^; dry temperature 325 °C; capillary voltage 3.5 kV. Analysis was carried out using scan from *m*/*z* 150–1000.

MC-HPLC-UV quantification experiments were carried out at the same chromatographic conditions with a UV-detection at 254 nm (iridoids) and 334 nm (flavonoids, mangiferin). An accurately weighted, dried, and powdered gentian herb tea samples (100 mg) was placed in a conical flask. Then 4 mL of 60% ethanol was added and the mixture was weighted. The sample was then extracted for 30 min at 40 °C in ultrasonic device UZV-2.8 (Sapfire, Moscow, Russia) with an ultrasound power of 100 W and frequency of 35 kHz, equipped with a temperature controller and a digital timer. After cooling, the flask weight was reduced to initial sign, and the resultant extract was filtered through a 0.22-μm PTFE syringe filter before injection into the HPLC system for analysis. Stock solutions of standards were made by accurately weighing 1 mg of loganic acid, loganic acid-6′-*O*-β-d-glucoside, swertiamarin, sweroside, gentiopicroside, isoorientin, isoorientin-4′-*O*-glucoside, isovitexin, saponarin, isosaponarin, isoscoparin, and mangiferin and dissolving it in 20 mL of methanol/DMSO in a volumetric flask. The appropriate amounts of stock solutions were diluted with methanol in order to obtain standard solutions containing 0.25–1.00 mg/mL. As all the compounds used for quantification were well-separated in experiment conditions mixtures of standards were analyzed. Prepared solutions were stored at 4 °C for no more than 72 h. The results are presented as mean values ± SD (standard deviations) of the three replicates.

### 3.6. Influence of Gentian Herbal Decoctions and Individual Compounds on Gastric Secretion

The experimental procedures relating to the animals were authorised by the Institute of General and Experimental Biology’s Ethical Committee (protocol No LM-0324, 27.01.2012) before starting the study and were conducted under the internationally accepted principles for laboratory animal use and care. Male rats (Sprague Dawley; six weeks old) were purchased from the “Pushchino” Laboratory Animal Breeding House, Moscow. Rats weighing *ca*. 200–270 g (seven weeks old) were used for the study. The animals were kept under standard pathogen-free conditions at a constant temperature (25 °C), humidity (55%) on a 12 h light/dark cycle, and were fed food and water *ad* *libitum*. The experiments were performed using 10–12 rats per group after more than 24 h fasting. Pylorus ligation was performed by the method of Shay *et al.* [39] with slight modifications as recorded by Kurasawa *et al.* [40]. The rats were treated with solutions of gentian decoctions in 0.9% NaCl (100 mg/kg) or individual compounds in 1% Tween-80 aqueous solution (20 mg/kg), while the control group was given an equal volume of 0.9% NaCl or 1% Tween-80 aqueous solution. All the treatments were administered orally. Rats were under anaesthesia about 3 min before pylorus ligation, and awoke within 15 min after the ligation. The abdomen of the rats was incised and the stomach and duodenum were exposed. The pyloric portion of the stomach was gently taken out and occluded with a ligature. At 0–4 h after pylorus ligation, rats were sacrificed, the stomach was removed, and gastric contents were collected. The gastric contents obtained from pylorus-ligated rats were centrifuged at 3000 *g* for 10 min, and the volume of each sample was measured as gastric juice volume. The sample was re-centrifuged for 10 min at 3000 *g*, and used for measuring free [41] and total HCl concentration [42], pepsin activity [43] and mucin concentration [44].

### 3.7. Statistical Analysis

Statistical analyses were performed using a one-way analysis of variance (ANOVA), and the significance of the mean difference was determined by Duncan’s multiple range test. Differences at *p* < 0.05 were considered statistically significant. The results are presented as mean values ± SD (standard deviations) of the three replicates.

## 4. Conclusions

The results of this study confirm the expressed potential of the four gentian plants for the production of bioactive compounds, and validate the ethnomedical use of the herb of *Gentiana algida*, *G. decumbens*, *G. macrophylla* and *G. triflora* as useful nutritional appetizers. Generally, chemical profiles of the mentioned gentian herbs as well as their gastric stimulant activity have not been previously reported. Our current findings demonstrated a scientific rationale for the use of gentian decoctions as a source of bioactive iridoids and phenolics (flavonoids, mangiferin). Also, the studied plants were proved as remedies for the treatment of gastric disorders (hypoacidity). With regard to general recommendations, it could be claimed that the gentian decoctions might be explored in the medicine industries.
